# Effect of albumin administration on outcomes in hypoalbuminemic patients hospitalized with community-acquired pneumonia (ALBUCAP): a prospective, randomized, phase III clinical controlled trial—a trial protocol

**DOI:** 10.1186/s13063-020-04627-1

**Published:** 2020-08-20

**Authors:** Alexander Rombauts, Gabriela Abelenda-Alonso, Antonella Francesca Simonetti, Guillermo Verdejo, Yolanda Meije, Lucia Ortega, Mercedes Clemente, Jordi Niubó, Yolanda Ruiz, Carlota Gudiol, Cristian Tebé, Sebastian Videla, Jordi Carratalà

**Affiliations:** 1 Department of Infectious Disease, Hospital Universitari de Bellvitge-Bellvitge Biomedical Research Institute (IDIBELL), Carrer de la Feixa Llarga, s/n, L’Hospitalet de Llobregat, 08907 Barcelona, Spain; 2 Department of Internal Medicine, Hospital Residència Sant Camil-Consorci Sanitari del Garraf, Sant Pere de Ribes, Barcelona, Spain; 3 Infectious Diseases Unit - Internal Medicine Department, Hospital de Barcelona, Societat Cooperativa d’Instal·lacions Assistencials Sanitàries (SCIAS), Barcelona, Spain; 4 Department of Microbiology, Hospital Universitari de Bellvitge, Hospitalet de Llobregat, Barcelona, Spain; 5 Department of Pneumology, Hospital Universitari de Bellvitge, Hospitalet de Llobregat, Barcelona, Spain; 6 University of Barcelona, Barcelona, Spain; 7 REIPI (Spanish Network for Research in Infectious Disease), Instituto de Salud Carlos III, Madrid, Spain; 8 Biostatistic Unit at IDIBELL, Barcelona, Spain; 9 Department of Clinical Pharmacology, Hospital Universitari de Bellvitge, Hospital de Llobregat, Barcelona, Spain

**Keywords:** Community-acquired pneumonia, Albumin, Inflammation, Randomized controlled trial

## Abstract

**Background:**

Community-acquired pneumonia (CAP) remains a leading cause of death worldwide, and hypoalbuminemia is associated with worse outcomes. However, it remains uncertain whether albumin administration could have any beneficial effects. We aim to assess whether the administration of albumin in hypoalbuminemic patients with CAP increases the proportion of clinically stable patients at day 5 compared with the standard of care alone.

**Methods:**

This is a trial protocol for a superiority, non-blinded, multicenter, randomized, phase 3, interventional controlled clinical trial. The primary endpoint will be the proportion of clinical stable patients at day 5 (intention to treat), defined as those with stable vital signs for at least 24 h. The secondary endpoints will be time to clinical stability, duration of intravenous and total antibiotic treatment, length of hospital stay, intensive care unit admission, duration of mechanical ventilation and vasopressor treatment, adverse events, readmission within 30 days, and all-cause mortality. The trial has been approved by the Spanish Medicines and Healthcare Products Regulatory Agency. The investigators commit to publish the data in peer-reviewed journals within a year of the study completion date. Subjects will be recruited from three Spanish hospitals over a planned enrolment period of 2 years. A follow-up visit will be performed 1 month after discharge. We have estimated the need for a sample size of 360 patients at a two-sided 5% alpha-level with a power of 80% based on intention to treat. Eligible participants must be hospitalized, hypoalbuminemic (≤ 30 g/L), non-immunosuppressed, adults, and diagnosed with CAP. They will be randomly assigned (1:1) to receive standard care plus albumin (20 g in 100 mL) every 12 h for 4 days or standard care alone.

**Discussion:**

If this randomized trial confirms the hypothesis, it should lead to a change in current clinical practice for the management of hypoalbuminemic patients with CAP.

**Trial registration:**

European Clinical Trials Database (EudraCT) 2018-003117-18. Registered on 12 April 2019. ClinicalTrials.gov NCT04071041. Registered on 27 August 2019

## Background

Community-acquired pneumonia (CAP) is an important public health problem [1]. This is underlined by an annual incidence of approximately 6.49 per 1000 inhabitants and a 10-fold rise in certain subgroups, such as the elderly and patients with chronic obstructive pulmonary disease [2]. Lower respiratory tract infection, and CAP in particular, is a significant cause of morbidity and mortality and remains a leading cause of death in high-income countries [3, 4].

Despite a decrease in short-term mortality among adult patients hospitalized with CAP over recent years, about 10% of these patients continue to die [2, 5, 6], with this figure rising to 28% in patients admitted to intensive care units (ICU), even when antibiotic treatment is adequate [7]. CAP has a significantly higher long-term mortality rate than many other conditions [8], occurring in nearly 1 in 3 adults 1 year after hospitalization [2]. CAP may also trigger acute cardiac events, further increasing morbidity, length of hospitalization, and mortality [9, 10]. Unsurprisingly, therefore, the economic burden of CAP is high, with an estimated yearly cost of 10.1 billion euros in Europe [11] and 13.4 billion dollars in the USA [12]. Inpatient care accounts for most of this cost, with length of stay, which is linked to a longer time to clinical stability, being the most significant factor. Longer stays also increase the risks of complications, such as phlebitis, ulcers, adverse drug reactions, and nosocomial infection [13].

The progress of patients hospitalized with CAP is crucial in the first days of therapy. In fact, once clinical stability is achieved, the course of infection is usually favorable [14]. Several studies have demonstrated that an inadequate inflammatory response is a major cause of early treatment failure and mortality [15–17]. Hypoalbuminemia also occurs often and is associated with poor outcomes in acutely ill patients, but it is unclear if this association is causal [18, 19]. A meta-analysis of 90 cohorts and 9 prospective controlled studies indicated that hypoalbuminemia was an independent predictor of poor outcome [18]. Hypoalbuminemia at admission is associated with higher mortality in severe sepsis [19] and with worse outcomes in patients with CAP [20–23], including a prolonged time to clinical stability and a longer hospitalization stay [22].

In humans, albumin accounts for about 50% of all plasma protein, while its molecular structure and concentration make it responsible for about 80% of the intravascular oncotic pressure [24]. Albumin metabolism in hospitalized patients is complex, and the pathophysiology underlying the relationship between plasmatic concentrations and ill-health is poorly understood. We know that stress and infection are associated with hypoalbuminemia, with an increase in albumin clearance, rather than decreased albumin synthesis, being dominant [25, 26]. In fact, a higher rate of albumin synthesis is typically observed in patients in ICU [27] and with acute inflammatory abdominal disease [28], and in healthy volunteers within 3 h after endotoxin administration [29].

It is now well accepted that, besides a role in maintaining colloidal osmotic pressure, albumin has secondary functions that are critical to normalizing many of the inflammatory pathways involved in sepsis [30]. Albumin transports many compounds and is responsible for most drug-protein binding, including antibiotics. Hypoalbuminemia can therefore lead to significant pharmacokinetic variation, such as an increased volume of distribution and an increased clearance of antibiotics [31]. Albumin also exerts specific antioxidant functions due to its multiple ligand-binding capacities, with two thirds of extracellular albumin existing in its reduced form. The reduced cysteine-34 (Cys34) residue accounts for 80% of plasma thiols, allowing albumin to trap reactive oxygen species, nitric oxygen, other reactive nitrogen species, and prostaglandins [32]. Moreover, albumin can bind several bacterial products [33]. In an endotoxin animal model, human albumin treatment was shown to have a dose-dependent protective effect on endothelial dysfunction through the inhibition of inflammatory and oxidative stress pathways [34]. Commercially available albumins differed in some of their antioxidant properties, primarily due to different levels of free Cys34 [35]. Our knowledge of the immunomodulatory effects of albumin is derived from in vitro and animal studies. Albumin enhances the ability of antigen-presenting cells to trigger T cell activation by increasing major histocompatibility complex II and human leukocyte antigen-DR expressions [36]. In fact, by binding prostaglandin E2, albumin was able to restore immune competence in patients with decompensated end-stage cirrhosis [37]. Two recent clinical trials demonstrated improved survival and a reduction in spontaneous bacterial peritonitis and other bacterial infections in cirrhotic patients with ascites [38, 39]. In endotoxemic rats, albumin resuscitation improved ventricular dysfunction by improving myocardial hypoxia and decreasing hypoxia-inducible factor (HIF)-1α expression [40].

To date, studies assessing albumin administration have focused on heterogeneous populations of septic and critically ill patients. A large randomized clinical trial evaluating the effect of volume replacement with human albumin versus crystalloid in 7000 critically patients failed to show a survival benefit, but decreased mortality was shown in a predefined subanalysis of patients with severe sepsis [41]. Consequently, the randomized controlled ALBIOS trial studied the impact of albumin administration in severe sepsis and septic shock, and although this also showed no mortality benefit, a post hoc subanalysis suggested that there was a lower 90-day mortality in the albumin group with septic shock [42]. Several systematic meta-analyses of albumin therapy in sepsis or septic shock have shown trends toward 90-day reductions in mortality associated with septic shock [43, 44], especially when administered within 6 h [45]. Other studies did not find any benefit [46–48]. However, it should be noted that these included different populations, comparators, and durations. This evidence led to the weak recommendation and low quality of evidence for using albumin in addition to crystalloids in the initial resuscitation of sepsis and septic shock in the surviving sepsis guidelines [49]. Although two new trials, ARISS (NCT03869385) and ALBIOSS-BAL (NCT03654001), seek to assess the impact of albumin administration in septic shock, no randomized trial has either been conducted or planned to analyze the effect of albumin treatment in patients with CAP.

We designed the current randomized multicenter trial to assess whether standard care plus albumin therapy will increase the proportion of clinically stable patients at day 5 of admission compared to standard care alone in patients with CAP and hypoalbuminemia.

## Design

### Study design and setting

This publicly funded academic study will be a non-blinded, multicenter, randomized, parallel, phase 3, interventional controlled clinical trial into the superiority of albumin plus standard therapy over standard therapy alone. The trial is sponsored by Dr. Jordi Carratalà—Hospital de Bellvitge—and began on November 14, 2019, and will run to November 2021. Patients will be recruited in three hospitals in Barcelona, Spain (Bellvitge University Hospital, SCIAS Hospital of Barcelona, and the Hospital Sant-Camil-Consorcio Sanitario Garraf). All items from the WHO Trial Registration Data Set have been registered at the publicly accessible databases ClinicalTrials.gov (NCT04071041) and European Clinical Trials Database (EudraCT 2018-003117-18). Informed consent will be obtained from all patients or their next of kin by the principal and subinvestigators.

### Study population

All hospitalized patients with CAP and hypoalbuminemia at presentation will be assessed for eligibility within 24 h of hospital admission.

The inclusion criteria are as follows:

- Age ≥ 18 years
- Diagnosis of CAP (Table 1)
- Serum albumin concentration ≤ 30 g/L at presentation

**Table 1:** Definition of community-acquired pneumonia

• Chest radiography consistent with community-acquired pneumonia	
• The presence of ≥ 2 following pre-specified clinical criteria:	
- Fever or hypothermia	
- Cough	
- Purulent sputum	
- High white blood cell count	
- Dyspnea	
- Pleuritic chest pain	
- Signs consistent with pneumonia on chest auscultation	

The exclusion criteria are as follows:

- Pregnancy or lactation
- Immunosuppression, such as chemotherapy or radiotherapy (< 90 days), immunosuppressive drugs, corticosteroids within 2 weeks of enrolment (≥ 15 mg/day of prednisone or equivalent), HIV-positive (CD4 count < 200), and solid organ or hematopoietic cell transplant recipients
- Severe clinical status with expected survival ≤ 24 h
- Congestive heart failure (New York Heart Association classes 3 or 4)
- Any contraindication for albumin administration, such as hypersensitivity to albumin
- Clinical conditions with an indication for albumin (e.g., hepatic cirrhosis with ascites, malabsorption syndrome, or nephrotic syndrome)
- Absence or impossibility of obtaining informed consent from patient or next of kin
- Patient already included in another clinical trial for a treatment method

### Patient and public involvement

When designing the study, our first priority was patient well-being. However, we have not included either patients or the public in the design, recruitment, or conduct of the study. Participants can request information about the study results through the principal investigator at any time.

### Randomization and allocation concealment

The Biostatistics Unit at IDIBELL will generate a centralized pre-specified block randomization list. The program will assign patients on a 1:1 basis to either standard care plus albumin or standard care alone. The block size will be ten, and participants will be stratified by center. The allocation list will be stored at the Biostatistics Unit.

### Intervention

Patients in the intervention group will be randomly allocated to receive standard care plus albumin 20% (20 g in 100 mL; Albutein Instituto Grifols, S.A. Can Guasch 2, Parets del Vallès, 08015 Barcelona, Spain) intravenously every 12 h for 4 days or until death, discharge, or clinical stability, whichever occurs first. Patients enrolled in the study will not require extended hospitalization or additional follow-up. Patients will receive empirical antibiotic therapy according to the relevant guidelines, as soon as CAP is confirmed. All microbiological assessments and additional treatments (e.g., oxygen, bronchodilators, corticosteroids, analgesics, vasoactive agents, fluid resuscitation, and mechanical ventilation) will be at the discretion of the treating physicians in both groups. The time of discharge and the duration of antibiotic therapy will not be determined by the study investigators, but by the treating team.

### Primary endpoint

The primary endpoint will be the proportion of clinically stable patients at day 5 of hospital admission. Clinical stability will be defined as previously described by Halm et al. as achieving normal oral intake, normal mental status (or usual level of function), and stable vital signs for at least 24 h (Table 2).

**Table 2:** Definition of clinical stability (Halm et al. 1998) [14]

• Temperature ≤ 37.8 °C without antipyretic agents	
• Heart rate ≤ 100 per minute	
• Spontaneous respiratory rate ≤ 24 per minute	
• Systolic blood pressure ≥ 90 mmHg without vasopressor support	
• Adequate oxygenation on room air (PaO_2_ ≥ 60 mmHg or pulse oximetry ≥ 90%)^a^	

^a^For patients with chronic hypoxemia or chronic oxygen therapy, PaO_2_, or pulse oximetry measurement, must be back to baseline

### Secondary endpoints

The following secondary endpoints will be assessed:

- Time to clinical stability (days) measured from hospital admission
- Duration of intravenous and total antibiotic treatment (days)
- Length of hospital stay (days) 
- ICU admission (time to discharge and durations of treatment with vasopressors and mechanical ventilation)
- Nosocomial infection during hospitalization
- Adverse events
- Hospital readmission within 30 days after discharge
- All-cause mortality at 5 and 30 days, as well as 30 days of discharge

### Stop treatment criteria

Comorbidities of CAP will not be considered sufficient to withdraw a patient from the study. However, the following stop criteria will be applied:

- Any unexpected serious adverse event between day 0 and day 5
- Clinical judgment of an attending physician or study investigator
- Voluntary withdrawal of consent
- Serious protocol violation
- Loss to follow-up
- Pregnancy during the study
- Congestive heart failure (New York Heart Association classes 3 or 4).

### Follow-up and data collection

**Fig. 1:**
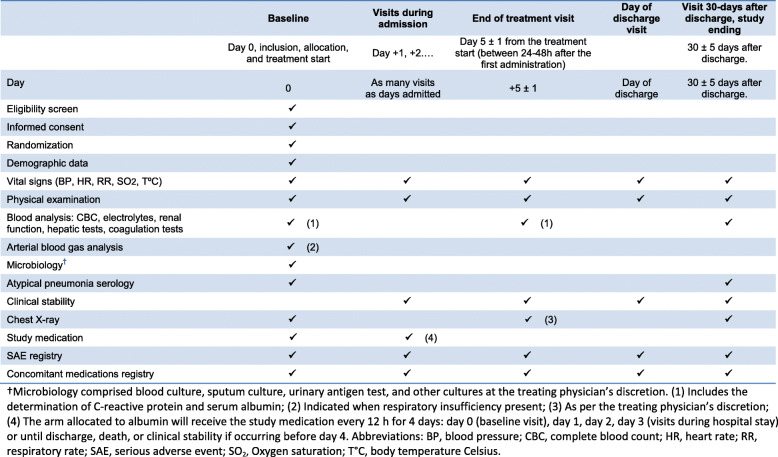
Participant timeline

All included patients will be daily assessed by a member of the investigating team until discharge or death. Consistent with routine clinical practice, a follow-up visit will be arranged for all participating patients 1 month after discharge. For patients who do not attend follow-up or in case of mobility restrictions in response to the COVID-19 pandemic, a structured telephone interview will be done to assess outcomes. A summary of the trial visit schedule and associated assessments is displayed in Fig. 1. Baseline data will include the following: date and time of randomization, demographic data, medical history items, relevant comorbidities, clinical features, causative organisms, antibiotic susceptibilities, antibiotic treatments, other medications, biochemical and microbiological analysis, and variables for the pneumonia severity index (PSI). Albumin concentration will be determined on days 0 and 5. Medication dosages will be recorded, and adverse events will be monitored appropriately throughout the study. In-hospital complications and cardiac events will be assessed. Any additional diagnostic testing in either group will be at the discretion of the treating physician. All data will be recorded on a secure web application used for building and managing online databases (REDCap). The sponsor and investigator will have access to the final trial dataset. Authorized staff may examine the records for quality assurance and audit purposes.

## Statistical analysis plan

### Sample size and power calculations

This study is designed to show the superiority of the intervention group (standard care plus albumin) at a two-sided 5% alpha-level with a power of 80% based on intention to treat. According to our own experience, the prevalence of hypoalbuminemia is 37% in patients presenting with CAP, with a predicted mortality rate of 10% [22]. We predicted that 58% of participants would be clinically stable at day 5 in the standard care group, with a difference of 15% between the two treatment arms. Given a possible deviation of 10% from the protocol, we will need a sample of 360 patients (180 per arm) to achieve a statistical power of 80% and a 5% significance level (two-sided). We aim to screen 900 patients.

### Type of analysis

We will use IBM SPSS version 21.0 (IBM Corp., Armonk, NY, USA) and R for Windows (R Foundation for Statistical Computing, http://www.r-project.org) for data analysis.

The primary analysis population is the full data set that includes the intention to treat population (all randomized patients). Every effort will be made to minimize the number of losses to follow-up. A secondary analysis will be conducted in a per-protocol population, excluding patients not treated according to the protocol. For sensitivity analysis, the primary analysis will then be repeated in the per-protocol population and by multivariable *t* test model (adjusted for potentially important confounders, such as age and PSI score).

We also plan to investigate whether the treatment effect varies between different CAP severities, between patients with and without chronic cardiac disease, and between patients with and without bacteremia. We will conduct the pre-specified subgroup analyses by including appropriate interaction terms in the multivariable *t* test. Complete case analyses will be used for secondary endpoints, for which we will calculate both unadjusted and adjusted estimates of the effect size and corresponding 95% confidence intervals by linear, logistic, or Cox proportional hazards regression.

The trial will be open, but the statistical analyst will be blind to the intervention and control group allocation.

### Interim analysis

To ensure sufficient statistical power, the sample size will be recalculated once half of the initial study population (180 patients) has been recruited. The necessity to increase the initial estimated sample size will be considered based on any variabilities encountered.

## Monitoring

Data will be monitored by a Data Monitoring Committee from the IDIBELL Clinical Research and Clinical Trials Unit. This committee is co-funded by the European Union and the Instituto de Salud Carlos III, Spanish Ministry of Science, Innovation and Universities, and it is independent of the sponsor.

## Adverse event reporting

An independent safety monitoring committee will review safety data. Any adverse event and its possible relationship to the study drug will be noted in patients’ clinical records. All severe adverse events (including death), and any related adverse events, will be recorded on the electronic case report form according to the Common Terminology Criteria for Adverse Events. An adverse event will be considered related to the study medication whenever a temporal relationship suggests a reasonable causal connection and/or the occurrence cannot be better explained by factors like the clinical status or another therapeutic intervention. The investigators will declare any serious adverse events to the sponsor within 24 h, and we will present a yearly security update report to the local regulatory agency consistent with the recommendations of the International Council for Harmonisation (ICH E2F).

## Indemnities

This study is classified as a low-intervention trial. According to Spanish law (Real Decreto 1090/2015), all damages incurred should be covered by the civil liability insurance of the participating study centers. An ad hoc insurance for the SCIAS Hospital of Barcelona (a private institution) has been taken out.

## Dissemination

The results will be presented at national and international meetings. The investigators commit to publish the data in peer-reviewed journals within a year of the study completion date. The datasets generated during the study will be available from the corresponding author on reasonable request.

## Protocol amendments

Relevant protocol modifications will not become effective until approved by the relevant authorities and the Drug Research Ethics Committee. Exceptions are changes to protect patients from imminent harms or exclusively concerning logistic and administrative aspects.

A protocol amendment adding pneumonia caused by SARS-CoV-2 to the exclusion criteria has been submitted and is currently pending decision from the relevant authorities and the Drug Research Ethics Committee.

## Discussion

Hypoalbuminemia is associated with worse outcomes in both sepsis and CAP [19–23]. To date, studies evaluating the efficacy of albumin therapy have been performed in heterogeneous populations of critically ill and septic patients. As such, they have rendered conflicting results [41, 42], although some have indicated clear benefits, notably in post hoc subgroup analyses [43]. We hypothesize that albumin administration in hypoalbuminemic patients with CAP could improve both the immune response and degree of organ dysfunction, thereby reducing the length of hospitalization and the time to clinical stability. This open-label randomized trial is designed to test this hypothesis, and it is expected that the results will determine if the addition of albumin therapy to standard care improves clinical outcomes. Even if the results are negative, we can confirm that there is no justification to change current clinical practice. However, if our hypothesis is proven, our study results could lead to a major change in practice.

## Trial status

The present manuscript describes the current authorized protocol at submission: version 1.1, 28 March 2019. The first patient was recruited on 14 November 2019, and enrollment is planned to run to November 2021. Recruitment was temporally halted from 14 March 2020 to 7 June 2020 due to the SARS-CoV-2 pandemic.

## Data Availability

Not applicable

## Abbreviations

BP: Blood pressure
CBC: Complete blood count
CAP: Community-acquired pneumonia
EudraCT: European Clinical Trial Database
HIV: Human immunodeficiency virus
HR: Heart rate
IDIBELL: Bellvitge Biomedical Research Institute
ICU: Intensive care unit
PSI: Pneumonia severity index
RR: Respiratory rate
SAE: Serious adverse event
SO_2_: Oxygen saturation
T°C: Body temperature Celsius
